# Molecular Epidemiology of *Staphylococcus aureus* Lineages in Wild Animals in Europe: A Review

**DOI:** 10.3390/antibiotics9030122

**Published:** 2020-03-14

**Authors:** Vanessa Silva, José L. Capelo, Gilberto Igrejas, Patrícia Poeta

**Affiliations:** 1 Microbiology and Antibiotic Resistance Team (MicroART), Department of Veterinary Sciences, University of Trás-os-Montes and Alto Douro (UTAD), 5000-801 Vila Real, Portugal; vanessasilva@utad.pt; 2 Department of Genetics and Biotechnology, University of Trás-os-Montes and Alto Douro (UTAD), 5000-801 Vila Real, Portugal; gigrejas@utad.pt; 3 Functional Genomics and Proteomics Unit, University of Trás-os-Montes and Alto Douro (UTAD), 5000-801 Vila Real, Portugal; 4 Associated Laboratory for Green Chemistry (LAQV-REQUIMTE), University NOVA of Lisboa, Lisboa, 2829-516 Caparica, Portugal; 5 BIOSCOPE Group, LAQV@REQUIMTE, Chemistry Department, Faculty of Science and Technology, NOVA University of Lisbon, 2825-466 Almada, Portugal; jlcm@fct.unl.pt; 6 Proteomass Scientific Society, 2825-466 Costa de Caparica, Portugal

**Keywords:** *Staphylococcus aures*, MRSA, *mec*C, wildlife, wild animals, epidemiology

## Abstract

*Staphylococcus aureus* is an opportunist pathogen that is responsible for numerous types of infections. *S. aureus* is known for its ability to easily acquire antibiotic resistance determinants. Methicillin-resistant *S. aureus* (MRSA) is a leading cause of infections both in humans and animals and is usually associated with a multidrug-resistant profile. MRSA dissemination is increasing due to its capability of establishing new reservoirs and has been found in humans, animals and the environment. Despite the fact that the information on the incidence of MRSA in the environment and, in particular, in wild animals, is scarce, some studies have reported the presence of these strains among wildlife with no direct contact with antibiotics. This shows a possible transmission between species and, consequently, a public health concern. The aim of this review is to better understand the distribution, prevalence and molecular lineages of MRSA in European free-living animals.

## 1. Introduction

Since the discovery of antibiotics, millions of lives have been saved, which has contributed to the average life expectancy of human beings increasing by 23 years [1]. However, the efficiency of these drugs has been surpassed by the resistance acquired by microorganisms, which leads the pathogen to cease to be susceptible to the antimicrobial agent. Antibiotic resistance can occur through a natural selection process, in which resistant bacteria remain, even in the presence of the antibiotic, reproducing and thriving [2]. The problem of antibiotic resistance was first discussed in public in the early 1940s, where overuse of antibiotics was discouraged [3]. However, in many countries these drugs are still available without a prescription [4]. The European Center for Disease Prevention and Control (ECDC) estimated that, in 2018, the consumption of broad-spectrum antibiotics in the European Union was 10.1 defined daily doses per 1,000 inhabitants in a single day [5]. Furthermore, still according to ECDC, 33,000 deaths occur in the European Union due to antibiotic resistant bacterial infections every year [5]. Although antimicrobial resistance (AMR) has always existed, the overuse and misuse of antibiotics have triggered an increase of antibiotic resistance strains. For instance, the enzyme penicillinase was detected in *Staphylococcus aureus* strains shortly after the introduction of penicillin, evidencing that the consumption of antibiotics will eventually favor the selection of resistant strains [6]. While antibiotics are progressively losing their effectiveness, life-threatening infections are becoming increasingly difficult to treat which entails high socioeconomic costs [7]. This situation is even more concerning, since antibiotics have become an essential element in procedures in modern medicine, such as in organ transplants [8].

Antibiotic resistance is a multifaceted problem and, even though more attention has been given to the use of antimicrobials in hospital settings, the use of antibiotics in animals, in particular farm animals, has recently gained some attention [9]. The administration of antibiotics to farm animals to promote growth has been banned in Europe since 2006; however, between European countries there is still a difference in the use of antibiotics in livestock, which suggests that the antibiotics use may exceed the actual therapeutic needs. In contrast, in other non-European countries, such as the USA, Canada and China, antibiotics are widely used to promote animal growth [10]. Additionally, the consumption of antibiotics in animal production is expected to increase 67% between 2010 and 2030, partly due to the great concern about the health and welfare of animals [11]. However, some microorganisms can infect both humans and animals and, therefore, the efforts of only one sector cannot prevent or eliminate the problem [12]. Antibiotic resistance and the emergence of zoonotic pathogens have increasingly threatened global health. As such, the concept of “One medicine” has been implemented recognizing that human and animal medicine can contribute to the development of one another [13]. Nevertheless, antibiotic resistance, which has emerged in clinical practices, is also found in animal production facilities, effluents and wastewater systems, thus becoming a problem involving not only humans and animals, but also the natural environment [14]. Moreover, microorganisms found in the environment converge with the pathogens of humans and animals, and the exchange of antibiotic resistance genes between bacterial strains from different environments can occur, thus dispersing through different routes: humans, animals, food and the environment [12]. Consequently, the “One Medicine” concept was not suitable, since it did not include a critical sector for the overall development of public and animal health, which are ecosystems and environmental health [12]. Thus, the “One Health” approach was institutionalized, which is based on the interaction between humans, animals and ecosystems in which they coexist [13]. While research has long focused on the role of healthcare facilities in the selection and spread of AMR, the potential role of the natural environment has been gaining attention only in recent years [15].

The term “ESKAPE” encompasses six pathogens with increased resistance to commonly used antibiotics: *Enterococcus* spp. vancomycin-resistant, methicillin-resistant *Staphylococcus aureus*, *Klebsiella pneumoniae, Acinetobacter baumannii, Pseudomonas aeruginosa*, and different Enterobacter species [16]. Therefore, there is a necessity to understand their resistance mechanism. In this review, we will focus on methicillin-resistant *Staphylococcus aureus.*

## 2. *Staphylococcus aureus*

The genus *Staphylococcus* belongs to the *Staphylococcaceae* family which includes 45 species and 24 subspecies, most of them being aerobic or facultative anaerobic [17]. *Staphylococcus aureus* are the main and most virulent species and received their name due to production of a carotenoid pigment which turns their colonies yellow on solid medium [18]. *S. aureus* are one of the seven species of coagulase-positive staphylococci (SCoP) identified so far that can cause severe infections when compared with those caused by coagulase-negative staphylococci [19]. *S. aureus* are also characterized by being catalase positive, oxidase negative, and salt tolerant [17]. They are frequently found on the skin and in the nasal mucosa of 25% to 30% of healthy people, where they live in an intimate relationship of commensalism or mutualism with the host [20]. In addition to the human being, *S. aureus* are also commensal organism in many homeothermic animals. However, these bacteria are opportunistic microorganisms recognized as a major cause of a variety of infections throughout history in both humans and animals [21]. Furthermore, when in adverse conditions, there is a breakdown of this usual balance, they may be able to cause infectious diseases in the hosts, becoming powerful pathogens with the ability to invade the bloodstream or internal tissues [22]. *S. aureus* can be responsible for a wide diversity of infections from skin and soft tissue infections to more serious infections, such as necrotizing pneumonia, endocarditis and osteomyelitis [22]. Despite an adequate treatment, *S. aureus* is one of the most frequent causes of bacteremia in humans, with a mortality, in a period of 30 days, of about 20% to 40% [23,24].

**Figure 1:**
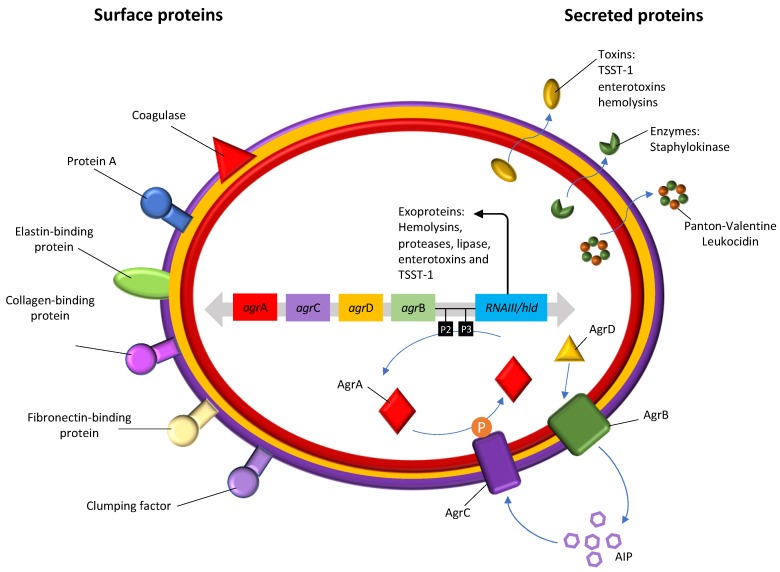
Regulation of virulence determinants in *S. aureus*.

These Gram-positive bacteria have several pathogenic mechanisms that make them effective in their ability to cause disease. *S. aureus* developed extracellular proteins and defense factors not associated with antibiotic resistance, which make it possible to evade the innate immune system [25]. Although *S. aureus* were initially classified as an extracellular pathogen, it is now known that they can be ingested by a large variety of eukaryotic cells persisting within these cells for long periods of time [26,27]. Inside the cell, *S. aureus* can persist inside the cells without causing inflammation and be protected against the action of antimicrobials which is one of the main reasons of antibiotic failure and relapse of *S. aureus* infection [28]. Furthermore, *S. aureus* may also be taken up by host phagocytic cells which contributes to disseminate these bacteria away from the initial site of infection leading to the invasion of other cells and tissues and, consequently, to chronic or recurrent infections [29]. Another reason for antibiotic failure in *S. aureus* infections is their ability to form biofilms. Due to biofilm matrix and phenotypic characteristics of the bacteria, biofilm formation impairs the action of antibiotics and also the host immune, being one of the most important survival mechanisms [28]. In bacterial biofilms the access of antibiotics to the deeper bacterial cells is hampered, thus decreasing their diffusion rates. In addition, the mechanisms of bacterial resistance to antibiotics in biofilms are altered, and cells normally susceptible to a given antibiotic, when they grow in biofilms, become quiescent, increasing their tolerance to that compound [30]. The production of the *icaADBC* operon-encoded polysaccharide intercellular adhesion is one of the most studied mechanisms of biofilm formation in *S. aureus* [31]. *S. aureus* also expresses several surface components which recognize adhesive matrix molecules facilitating the cell adhesion, thus playing an important role in *S. aureus* pathogenesis [32]. *S. aureus* produces extracellular enzymes and a wide range of closely coordinated and regulated virulence factors (Figure 1). The range of virulence factors in *S. aureus* is extensive and includes more than 40 proteins, including enzymes, which are secreted and used to establish and maintain infections [33]. Some of these virulence factors are known to cause or be associated with diseases such as the case of the thermostable proteins responsible for food poisoning, such as classic and non-classical staphylococcal enterotoxins; the superantigenic exotoxin, expressed in about 25% of *S. aureus* strains, responsible for the toxic shock syndrome (Toxic Shock Syndrome Toxin-1 (TSST-1)) when introduced into the bloodstream; Panton-Valentin leukocidin (PVL), which causes the destruction of leukocytes and tissue necrosis, and is often associated with a specific type of pneumonia called necrotizing pneumonia; exfoliative toxins A and B (ETA and ETB) that function as “molecular scissors” and facilitate the invasion of the skin by microorganisms, which leads to the appearance of impetigo, atopic dermatitis and other dermal infections; and the alpha, beta and delta hemolysins [34,35,36]. These last ones are the most studied and characterized of the cytotoxins produced by *S. aureus*, especially hla (alpha hemolysins), which is referred to as one of the main factors of pathogenicity of *S. aureus* [37]. The virulence factors, as well as part of the proteins on the *S. aureus* cell surface are regulated by a *locus*, which serves as a global regulator, the accessory gene regulator (*agr*), consisting of clustered genes. Polymorphisms in the *agr*B, *agr*C and *agr*D gene define four specific *agr* groups (groups I–IV) with different degrees of virulence. This pathogen, in addition to the metabolic diversity, also has a high capacity to acquire resistance to antibiotics, and is thus well adapted to various environmental circumstances, allowing the colonization of man and the environment around him [38].

### 2.1. Methicillin-Resistant S. aureus

*S. aureus* has the ability to acquire a variety of mechanisms of resistance to antimicrobial agents. The first antibiotic introduced in the market used in *S. aureus* infections was penicillin; however, in the late 1950s, resistance to penicillin found in *S. aureus* was a cause of huge concern [39]. To counteract the worldwide spread of penicillin-resistant *S. aureus*, methicillin β-lactam antibiotics and, later, oxacillin, were synthesized [39]. Nevertheless, shortly after the introduction of methicillin, methicillin-resistant *S. aureus* (MRSA) strains also emerged, becoming one of the most life-threatening antibiotic-resistant pathogens [40]. Resistance to methicillin is the result of two distinct mechanisms: the production of β-lactamases leading to decreased activity of β-lactam antibiotics and the production of penicillin-binding protein 2a (PBP2a) [41]. The PBP2a is an enzyme that actively participates in peptidoglycan synthesis, responsible for promoting resistance to bacterial cell walls but whose active site of binding to β-lactams is inaccessible, preventing its action and thus allowing the normal course of cell wall synthesis of bacteria [41]. This is encoded by the *mec* genes, while β-lactamases are encoded by the *bla*Z gene. The origin of *mec*A gene is not exactly known; nevertheless, some studies theorize that these resistant determinant originated from some species of coagulase-negative staphylococci (SCoN) since homologues of the *mec*A gene have been found in *S. sciuri* group species, which includes the *S. sciuri*, *S. lentus* and *S. vitulinus* species, suggesting that this group may be the evolutionary precursor of the *mec*A [42]. The *mec* genes are included in the staphylococcal cassette chromosome *mec* (SCC*mec*), which is a mobile genetic element of staphylococci. Several *mec* genes have been described so far. Two *mec*A homologues with 80% and 90% of similarity, classified in both cases as allotypes *mec*A1 and *mec*A2 were detected in *S. sciuri* subsp. *rodentium* and in *S. vitulinus*, respectively [43]. Other homologues, with less than 70% identity, have been reported—the *mec*B, *mec*C, and *mec*D genes; however, *mec*B and *mec*D were initially described in in *Macrococcus caseolyticus* [44,45]. More recently, *mec*B was detected in *S. aureus* strains, which suggests a possible transfer between genus [46]. Three *mec*C allotypes, *mec*C1, *mec*C2 and *mec*C3, were detected in *S. xylosu, S. saprophyticus* and *S*. *caeli* of milk, a small mammal and air sampling of a commercial rabbit [47,48,49]. The *mec*A gene was widely distributed among human MRSA isolates and it was not until 2011 that *S. aureus* isolates harboring the *mec*C gene was found in humans, livestock, companion and wild animals [50,51,52]. The first assumption was that the *mec*C was associated with LA-MRSA; however, the detection of *mecC*-MRSA in wild animals [51,53,54], wastewaters [55] and surface water [56] indicates that the primary reservoirs of *mec*C gene may be the natural environment.

**Figure 2:**
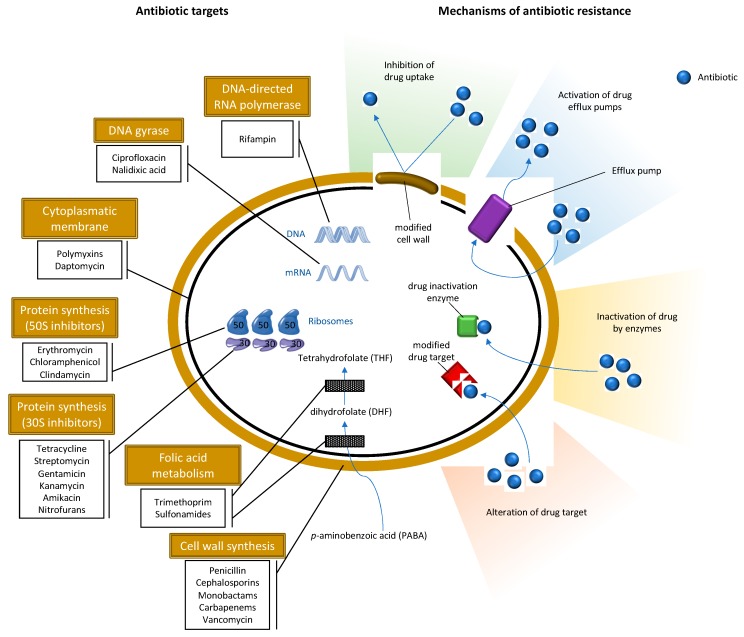
Schematic representation of antibiotic targets and mechanisms of antimicrobial resistance in *S. aureus*.

Besides being resistant to most β-lactam antibiotics, MRSA are frequently associated with resistance to other classes of antibiotics (Figure 2). The great majority of HA-MRSA (healthcare-acquired MRSA) strains are often resistant to non-β-lactam antimicrobial agents especially aminoglycosides, macrolides, lincosamides and fluoroquinolones, and resistance to all antibiotic classes has been described in MRSA although resistance to, for instance, vancomycin and linezolid are still uncommon [57]. Vancomycin has been the choice in the treatment of infections by this resistant pathogen [58]. However, in the late 1990s, MRSA strains with reduced susceptibility to vancomycin were isolated and, eventually, vancomycin-resistant *Staphylococcus aureus* (VRSA) strains were later detected [59]. Some HA-MRSA clones, usually associated with nosocomial infections, have spread to the community. Community-acquired MRSA (CA-MRSA) emerged in the late 1990s as major causes of skin and soft tissue infections in healthy people with no previous hospitalizations or recent histories of invasive procedures. CA-MRSA are newer and more virulent strains frequently associated with PVL which are associated with increased virulence [60]. CA-MRSA are distinct from HA-MRSA from an epidemiological, genotypic and phenotypic point of view. CA-MRSA have the ability to spread more easily and show higher fitness when compared to HA-MRSA. Furthermore, CA-MRSA strains belong to the SCC*mec* type IV, V or VI, whereas HA-MRSA usually belong to the types I, II or III [61]. The strains of CA-MRSA have been extensively studied and found in several animals with which humans have a direct contact, such as pets, cattle, pigs and horses, as well as in wild animals [62,63]. Since 2005, MRSA associated with farm animals started to emerge in the human population [64]. The first case of livestock-associated MRSA (LA-MRSA) was reported in early 1970, in Belgium, in bovine mastitis [65]. One of the most important strains of LA-MRSA corresponds to the molecular type ST398 that was initially found in pigs, then in several other animals, and later in humans mainly with professions that have frequently contact with these animals [62].

### 2.2. S. aureus Characterization by Molecular Typing

Molecular typing of bacteria is essential, both clinically and epidemiologically, to determine the cause of the infection, modes of transmission during outbreaks or to analyze their relationship with other bacteria, as well as to assess the specific characteristics of the genetic lineage. There is a wide range in terms of typing techniques (Table 1). For this reason, it is common to combine several methods, depending on the purpose of each study [66].

#### 2.2.1. Accessory Gene Regulator (*agr*) Typing

*S. aureus* has a quorum sensing system, the paradigmatic *agr* (accessory gene regulation) system, that is capable of regulating the adhesion and production of numerous virulence and pathogenicity factors as well as the biofilm formation and heterogeneous resistance of *S. aureus* [67]. The locus *agr* is a global regulatory system among staphylococci and it was first described in *S. aureus* in 1988 [68]. This operon is self-induced by a peptide (AIP) that is located in the same locus, diffuses into the target cell and acts as a ligand of the signal receptor triggering the generation of a cascade that induces the production of toxins [69]. The *agr* locus consists of four genes: *agr*B encodes a membrane protein responsible for translocation and modification of AgrD; *agr*D encodes an AIP precursor octapeptide; *agr*C encodes a membrane receptor protein of the AIP signal and *agr*A encodes the AgrA response regulator that activates transcription. *agr* varies between *S. aureus* strains and can be divided into four groups (*agr* type I, II, III and IV) [70]. Studies have reported the association between the *agr* types and the different clonal lineages, antibiotic resistance profile, biofilm formation, the toxins produced and their link with diseases [71]. For instance, *agr* type I and II strains are usually associated with endocarditis and septicemia whereas *agr* type III and IV strains are associated with TSST-1, exfoliative syndromes and bullous impetigo [70].

#### 2.2.2. *spa* Typing

*S. aureus* protein A (*spa*) is a virulence factor which prevents opsonization and phagocytosis. The X region of *spa* comprises repeats exhibiting an extensive polymorphism based on point mutations, deletions, duplications, and insertions. The amplification and sequencing of the 24 bp polymorphic zone X of the *spa* gene gives several repeats which can be assigned an alpha-numerical code and the *S. aureus* strain can be determined [72]. *Spa* typing has become a widely distributed typing technique for *S. aureus* since it is simple and cheap, with greater discrimination power when compared to MLST [73]. At the beginning of 2020, more than 19,000 different *spa*-types were registered in the Ridom SpaServer.

Studies have demonstrated that there is a fairly good correlation between the *spa* gene repeat polymorphism and MLST clonal groups. However, misclassification may occur when assuming that a strain belongs to an MLST lineage based on its *spa* type since isolates with similar *spa* profiles may belong to distant MLST clonal complexes [74].

#### 2.2.3. Multilocus Sequence Typing

Multilocus sequence typing (MLST) is a molecular technique developed in 1998 for the identification of clones of pathogenic bacteria [75]. It is based on sequence analysis fragments from seven housekeeping genes which are highly conserved, as they encode enzymes necessary for the metabolism of the bacteria. This technique was first used in 2000 for *S. aureus* with the sequencing of the following genes: *arc*C (carbamate kinase), *aro*E (shikimato dehydrogenase), *glp*F (glycerol kinase), *gmk* (guanylate kinase), *pta* (phosphate acetyltransferase), *tpi* (triosaphosphate isomerase) and *yqi*L (acetyl coenzyme A acetyltransferase) [76]. The sequence of each locus is assigned to an allele identification number based on its similarity with known alleles, and the combination of these seven alleles generates a type sequence (ST) [77]. STs are considered closely related and can be grouped under the same clonal complex (CC), with the software package BURST, when there are polymorphisms in a single nucleotide in less than three genes [78]. CA-MRSA mainly belong to clonal complexes CC8 (ST8), CC30 (ST30), CC59, CC80 and CC93; HA-MRSA are mainly assigned to CC5, CC8 (ST239), CC22, CC30 (ST36) and CC45; and LA-MRSA are mostly ascribed to CC398 [79]. Moreover, certain *S. aureus* clonal lineages correlate with different *agr* types: clonal lineages CC8, CC25, CC22, CC45, and CC395 are usually associated with *agr* type I; CC5, CC12, and CC15 with *agr* type II; CC30 is often characterized by *agr* type III, and CC121 by *agr* type IV [70].

#### 2.2.4. SCC*mec* Typing

Staphylococcal cassette chromosome *mec* is a mobile genetic element that carries the *mec* genes responsible for the β-lactam resistance. The dissemination of the methicillin resistance in staphylococci strains is due to acquisition and insertion of the SCC*mec* element into the chromosome of susceptible strains. SCC*mec* elements have been classified into types and subtypes and it is now common to define MRSA clones using the combination of CC or ST and SCC*mec* type [80]. The *mec* genes encode PBPs with lower affinity for β-lactams, and their expression is regulated by two genes, *mec*I and *mec*R1. *mec*I encodes a gene transcription repressor protein of the *mec* genes while *mec*R1 acts as a transcriptional regulator of gene expression. When in the presence of β-lactam antibiotics, the membrane protein MecR1, encoded by *mec*R1, recognizes the antimicrobial in its receptor domain and induces an autocatalytic protease that inactivates the repressor, thereby allowing transcription of the *mec* gene. SCC*mec* elements also contain the *ccr* (cassette chromosome recombinase) gene complex, which contain the *ccr*A, *ccr*B and *ccr*C genes, that encode recombinases capable of cleaving and integrating the entire SCC*mec* element. SCC*mec* types are defined by the *mec* and *ccr* gene complexes and, 13 different types of SCCmec (I-XI) are known in *S. aureus* to date, in addition to a variety of subtypes depending on the variations in the binding regions (J1-J3) (http://www.sccmec.org). The J regions are cassette components that may contain determinants for additional antimicrobial resistance; thus, within the SCC*mec* we can also find resistance genes to other non-β-lactam antibiotics such as erythromycin, clindamycin, tetracycline, aminoglycosides, clindamycin, among others [81]. Furthermore, there are also determinants of virulence and resistance to heavy metals within the SCC*mec* [82]. Currently, there are many strains that carry non-typed SCCmec elements or even with two types of *ccr* and or *mec* complex, and so there is still much to discover about the SCC*mec* system. Traditionally, there are 11 types of SCCmec; however, more recent studies have expanded the classification with two new types, type XII discovered in 2015 [83] and type XIII more recently [84]. The *mec*C gene is located in the SCC*mec* type XI and is frequently detected in animals associated with CC130. Although CC130 is considered a livestock-associated MRSA, its repeatedly detection among wild animals and natural environmental compartments indicates otherwise [85].

## 3. Antibiotic Resistance in the Environment

The natural environment plays an important role as a reservoir and disseminator of AMR. Bacteria present in soil and water can act as reservoirs of antimicrobial resistance genes (ARG), which are a vector for the transmission of AMR [97]. Due to the intensive usage of antibiotics in medicine and agriculture, antibiotic-resistant bacteria present in the environment are more likely to be selected within polluted environments directly by selective pressure exerted by antibiotics or indirectly through co-selection by other contaminants [98,99]. Veterinary medicine and wastewater treatment plants are the major sources of release of human antibiotics, AMR and antimicrobial resistance genes (ARGs) into the natural environment, which has led to the selection of antibiotic resistant bacteria in the environment [99]. Selective antimicrobial pressure is particularly high in hospitals. In fact, about 20% to 30% of European patients have undergone antibiotic therapy during their hospitalization. In addition, hospitals are a very important source of the spread of pathogens, constituting ecological niches for antibiotic resistant bacteria [97]. These bacteria leave the hospital through colonized patients and also, through wastewater systems [100]. Thus, aquatic ecosystems provide an ideal scenario for the acquisition and spread of antibiotic resistance genes [101]. However, the spread of resistant bacteria is worsened when hospital wastewater is discharged directly into public sanitation networks without prior treatment [100]. Furthermore, the large amounts of antibiotics found in wastewater, may exert selective antimicrobial pressure. Heavy metals and disinfectants with antibacterial properties also favor the bacterial resistance present in these waters [100]. Surface waters are the main recipient of treated and untreated wastewaters favoring the dissemination of resistance genes in microbial communities via water, land or even through wildlife [102]. Nonetheless, only a few studies have been carried out in Europe in order to determine the presence of *S. aureus,* its antimicrobial resistance profiles, and its genetic lineages in surface waters [56,103,104,105]. These studies have found *S. aureus*, among other staphylococci, with low rates of antibiotic resistance, and *mec*A and *mec*C-carrying MRSA strains belonging to a wide diversity of clonal complexes: CC5, CC7, CC8, CC12, CC22, CC30, CC45, CC59, CC101, CC130, CC133, CC398, CC425, and CC707. On the other hand, livestock may also be a great source of antibiotics and AMR, since a great part of the antibiotics used in these animals are excreted with through urine and manure. When animal waste release into the environment on land application of manure reach the upper soil it will disturb the bacterial communities affecting the abundance, diversity, and transferability of ARGs, which may lead to the acquisition of gene-encoding resistance by soil bacteria [99]. Thus, anthropogenic sources of AMR and AGRs, such as wastewater systems, effluents, and animal husbandry facilities, are characterized by high bacterial loads combined with subinhibitory doses of antibiotics being a pool of AMR and AGRs discharged into the environment [106]. In addition to water and soil, another route of dissemination of antibiotic-resistant bacteria is the air and dust. Several studies have investigated the occurrence of MRSA in the air and on soil surfaces of pig barns. Most studies reported a low prevalence of LA-MRSA in air samples, nevertheless, the presence of MRSA in air samples may contribute to the spread of this strain in the natural environment [107,108,109,110].

Transmission of AMR between the natural environment to humans, and vice versa, may occur and, although it has not been possible to establish to what extent this occurs, there is evidence that the environment is a vast reservoir of antibiotic resistant bacteria and their resistance determinants [15]. Yet, this so-called “resistome” predates human use of antibiotics and is part of the natural microbial populations [111]. The “resistome” is the term used to define the ecology of resistance on a global scale, and consists of all antibiotic resistance genes found in pathogenic and non-pathogenic bacteria and also, in antibiotic producers [112]. Speculation states that antibiotic producers found in nature are the main source of resistance genes found in pathogenic bacteria [113]. Nevertheless, studies have shown that all bacteria possess genes that encode responses to small external molecules either for protection or nutrition [114]. One of the first studies regarding the soil resistome investigated a collection of 480 spore-forming bacterial strains from soil samples of several origins which were screened against 21 antibiotics, including natural products, semisynthetic derivatives and completely synthetic molecules that have been on the market for decades, as well as recently approved ones. All strains presented a multidrug-resistant phenotype, and resistance to all antibiotics was also detected. This study provided information of the soil resistome and the antibiotic resistance burden outside the clinic [115].

ARGs can be acquired from any source and transferred between bacteria corresponding to different phyla [116]. Their dissemination is usually associated with mobile genetic elements. Nevertheless, AGR flow is associated with the ecology of bacterial species that share similar niches and acquire their AGRs from similar gene pools [117]. The dissemination of ARGs through the environment can occur by several different mechanisms: chromosomal mutations, horizontal gene transfer and/or intracellular migration [118]. Initially, when the first resistances were reported, it was thought that these were due exclusively to mutations that occurred spontaneously, leading to the emergence of resistant organisms [119]. In this scenario, mutant cells derived from a susceptible population have the ability to survive even in the presence of the antibiotic, while susceptible ones will be eliminated. However, the discovery of horizontal gene transfer (HGT) processes and extra chromosomal DNA elements quickly put the theory of mutational resistance in the background [119]. Unlike vertical transfer, where genes pass from the mother cell to daughters during reproduction, horizontal gene transfer can result from the direct acquisition of external DNA released by neighboring cells, transmission of cell-to-cell DNA across the cell surface and virus-mediated DNA transfer. The mechanisms of antibiotic resistance can be intrinsic or acquired. Intrinsic resistance, also called natural resistance, results from a long process of genetic evolution and arises due to physical characteristics typical of the species. For example, *Pseudomonas aeruginosa* is naturally resistant to penicillins due to the antibiotic’s inability to cross the bacteria’s outer membrane and also due to the presence of β-lactamases. On the contrary, acquired resistance appears in a short period of time in a bacterial population that was already susceptible through changes in bacterial DNA. These changes are caused by chromosomal mutations, horizontal gene transfer or intracellular migration of resistance genes [120].

### S. aureus and MRSA in Wild Animals

Staphylococci have the ubiquity of surviving in adverse environmental conditions and can be found, in addition to on the skin and mucous membranes of animals and people, in the air, dust, water, soil, plants or environmental surfaces. Nevertheless, studies describing the prevalence, AMR and virulence, and genetic lineages of *S. aureus* in environmental niches are scarce. As mentioned above, people, animals and the environment are interconnected, and a constant flow of resistance genes and bacteria between different ecological niches and living things is possible. The ability of *S. aureus* to acquire antibiotic resistances along with its zoonotic potential highlights the importance of studying this microorganism in other contexts, such as in free-living animals. Studies have detected and characterized *S. aureus* in different wild animals, such as, among others, in hares, deer, foxes, mice, mountain goats, kangaroos, hedgehogs, bears, wild boars, beavers, squirrels, shrews, bats, minks, raccoons, seals, apes, as well as in different species of birds [121,122,123,124,125,126,127,128,129,130].

A very complete study conducted in 2016 by Monecke et al. analyzed the fecal samples of 2855 wild animals from Austria, Germany and Sweden [123]. From these, 155 *S*. *aureus* were isolated, of which 124 were further characterized, and they were assigned to 29 CCs as follows: CC1 (fox, fallow deer, raven, mouflon), CC5 (hare, partridge), CC6 (fox), CC7 (fox), CC8 (fox, mouflon, marmot), CC9 (wild boar), CC12 (porpoise), CC15 (raven, elk), CC22 (raven, fox), CC25 (badger), CC30 (marmot, deer), CC49 (vole, cat), CC59 (wild boar), CC88 (crow), CC97 (eagle, wild boar, elk, roe deer), CC130 (fallow deer, hedgehog, fox, rat, hare, SARM and SASM), CC133 (swan, wild boar, roe deer, chamois), CC398 (hare, SARM), CC599 (hedgehog), CC692 (eagle, magpie, dove, owl, woodpecker, great tit), CC707 (reindeer), CC1956 (topillo), CC2767 (lynx, reindeer). Most isolates were methicillin-sensitive *S. aureus* (MSSA). This study shows that MSSA and MRSA strains isolated from wildlife have a great diversity, with clonal lineages associated with humans and animals, while others appear to be less common and unique, such as CC692. Studies regarding the presence of *S. aureus*/MRSA in European free-living animals will be further discussed in detail.

Studies have been carried out with respect to a variety of European wild mammals (Table 2). In a study by Loncaric et al. (2013), 40 different wild animals were screened, and *S. aureus* was isolated from a European otter (*Lutra lutra*) and a European hedgehog (*Erinaceus europaeus*). Both isolates were methicillin-resistant and harbored the *mec*C gene. Regarding the clonal lineages, MRSA from the hedgehog belonged to CC130 and *spa*-type t3256, and the strain isolated from the otter was ST2620 and *spa* t4335 [51]. Nowakiewicz et al. (2016) screened a total of 76 wild animals, namely, red fox (*n* = 39), northern white-breasted hedgehog (*n* = 3), European polecat (*n* = 1), European pine marten (*n* = 24), roe deer (*n* = 5), serotine bat (*n* = 1) and European hamster (*n* = 3). MSSA were detected in two foxes, three European pine marten and one hedgehog, and showed resistance to erythromycin and clindamycin, which was characterized by the presence of the *erm*A and *erm*B genes. In the same study, one MRSA isolate was obtained from the marten. This isolate harbored the *mec*A gene and carried the genes *bla*Z, *msr*A, *tet*K and *tet*M, responsible for resistance to ampicillin, cefotaxime, erythromycin and tetracyclin. Further characterization showed that the MRSA isolate was ST8, *spa*-type t1635 and PVL positive [126]. The carcasses of 242 alpine wild ruminants, 276 foxes, 134 mustelids and 16 rodents were analyzed by a team in Italy, and *S. aureus* was found in one rodent, eight ruminants, one marten and one fox; nevertheless, the molecular analysis of the isolates was not performed [131]. A more recent study conducted with 103 free-living mammals from Spain detected 23 *S. aureus*. Positive samples included 11 wild boars, 4 red deer, 4 mouflons, 3 rabbits and 1 hedgehog. Among the 23 *S. aureus*, four MRSA were detected, being three *mec*C-MRSA from three wild rabbits and one *mec*A-MRSA from the hedgehog. The *mec*C-MRSA were typed as t843 (ascribed to CC130) and showed resistance only to β-lactams, whereas the *mec*A-MRSA showed resistance to penicillin, cefoxitin, erythromycin, streptomycin and inducible resistance to clindamycin and induced by the *blaZ*, *erm*(C), and *ant*(6)-Ia genes, and was typed as CC1 and *spa*-type t386. Regarding the MSSA isolates, 10 different *spa*-types were detected (t1125, t1534, t1535, t3750, t6056, t6386, t7174, t11225, t11230 and t11233). All isolates from this study were PVL-negative [54]. Porreto et al. (2013) screened 1342 animals including 273 red deer, 212 Iberian ibex, 817 wild boar and 40 Eurasian Griffon vultures. MRSA was only detected in one red deer, two ibex, two vultures and seven wild boars. Two MLST (CC398 and CC1) and three *spa*-types (t011, t1451and t127) were detected. Isolates also showed resistance to tetracycline, ciprofloxacin, erythromycin and clindamycin [132]. Nine native wildlife species, namely, four red squirrels, one white-tailed eagle, one red kite, one roe deer and one European beaver were positive for *S. aureus*. However, no isolates were resistant to methicillin. The four MSSA isolates from squirrels fell into three *spa*-types (t208, t307, t528) and three lineages (CC49, CC22, ST4310); the MSSA of the deer, eagle, kite, beaver and bat were t15473, t1422, t14745, t3058 and t164, and CC425, CC692, CC692, CC1956 and CC20, respectively [133].

Hedgehogs, a small nocturnal mammal, have been widely studied with respect to the carriage of *S. aureus* and MRSA, and some studies have reported a high occurrence of *mec*C-MRSA among these animals. Bengtsson et al. (2017) analyzed 55 hedgehogs’ samples, which were recovered at wildlife rescue centers in Sweden, and found a high occurrence (64%) of *S. aureus*. All strains were resistant to methicillin and all harbored the *mec*C gene. Most isolates had reduced susceptibility only to β-lactams, 7 presented resistance to other antimicrobial agents, and all isolates were PVL-negative. Eight different *spa*-types were identified (t843, t978, t3391, t9111, t10751, t10893, t11015, t15312); although MLST was not performed, according to spa type the isolates most likely belong to CC130 and CC2361 [121]. Another study conducted with hedgehogs analyzed 188 dead animals from a pool sample of 697 collected throughout Denmark. One hundred and fourteen individuals carried *mec*C-MRSA. As in the previous study, the genes encoding PVL were absent; however, all isolates were susceptible to all tested antimicrobials except the β-lactams. Most MRSA belonged to CC130 (*n* = 70) and CC1943 (*n* = 44) and 12 different *spa*-types were found (t528, t843, t1048, t3256, t3570, t6220, t17133, t978, t2345, t3391, t8835 and t16868) [134]. In a study by Monecke et al. (2013), two diseased free-ranging European hedgehogs presenting lesions related to infection were screened for the presence *S. aureus*. Both animals carried *mec*C-positive MRSA strains resistant penicillin and cefoxitin. MRSA isolates were typed as CC130, one was spa-type t843 and the other to t5771. Both isolates were PVL-negative [135].

In the last few years, the wild boar (*Sus scrofa*) population has been increasing in several European countries and it has been hypothesized that these animals could play an important role in the dissemination of several diseases [147]. Wild boars are one of the most studied wild animals in Europe and there are several studies investigating the *S. aureus* colonization of these animals. The meat of boars is widely appreciated and is considered a delicacy. Kraushaar and Fetsch (2014) conducted a study in 28 MRSA isolates from wild boar meat in Germany. All isolates carried the *mec*A gene. Other resistance genes were detected among the isolates, such as the *bla*Z gene conferring resistance to ampicillin–penicillin and the *msr*A and *mph*C genes (macrolide resistance). MLST was not carried out; however, 20 isolates comprised *spa* types related to the clonal complex CC398, namely, t011, t034, t1456 and t1250. Eight isolates were not related to CC398 and were typed as t015 (CC45), t202 (CC93) and t008 (CC8). Seven of the eight non-CC398 MRSA isolates were positive for the PVL genes [136]. In a more recent study also carried out in Germany, samples recovered from 111 wild boars were analyzed and 41 *S. aureus* isolates were obtained. All isolates were tested negative for virulence genes and for methicillin resistance. MSSA were ascribed to 19 different *spa* types (t127, t091, t14149, t021, t1773, t11226, t1181, t7674, t12042, t10856, t3369, t15002, t6902, t15001, t15000, t3583, t742, t14999, t571) and 11 STs (ST1, ST7, ST30, ST890, ST3237, ST3238, ST3369, ST425, ST3255, ST133, ST804) [138]. In a study conducted in Portugal, 45 wild boars were screened, and 30 *S. aureus* were isolated. Only one MRSA was found and was typed as ST398, and *spa*-type t899. It showed resistance to tetracycline and ciprofloxacin and harbored the *mec*A gene. The remaining 29 MSSA belonged to the sequence types ST3220, ST1, ST3224, ST3223 and ST3222, and *spa*-types t3750, t1533, t286, t14312, t14311, t10668, t3583, t3750, t11230 and t10712 [122]. Samples from 371 wild boars were collected in Spain. From those samples, 50 MSSA and 1 *mec*A-MRSA were recovered. Twenty-two different *spa* types and eight STs were detected among the MSSA. The only MRSA strain was ascribed to CC398 and *spa*-type t011, and showed resistance to penicillin and tetracycline, harboring the *bla*Z, *mec*A, *tet*(M) and *tet*(K) genes [137].

Small wild mammals, including rodents, may act as reservoirs of zoonotic pathogens and, therefore, may be implicated in public health risks. Nevertheless, research regarding the presence of MRSA in wild rodents revealed low rates of prevalence. A study carried out in samples from 101 wild rodents showed that only two *S. aureus* (out of 13 *S. aureus* isolates) carried the *mec*C gene. Both isolates were typed as *spa* t1535 and ST1945 (ascribed to CC130) and presented several virulence factors. Most of the MSSA isolates showed susceptibility to all tested antimicrobials. Five new *spa* types (t12363, t12364, t12365, t12752 and t12863) and two new STs (ST2766 and ST2767) were identified in among the MSSA strains [140]. Sixty one individuals from two rodent species *(Apodemus agrarius* and *A. flavicollis)* were screened for *S. aureus* in Slovakia. Seven strains of *S. aureus* were isolated, with three being resistant to methicillin and harboring the *mec*A gene [125]. Mrochen et al. (2018) studied 295 wild rodents and shrews. *S. aureus* were isolated from 45 animals. Five *S. aureus* from shrews belonged to t9909 and ST3033. Only one *mec*C-MRSA was found which was ascribed to CC130 and *spa*-type t843. The remaining MSSA strains fell into 10 different *spa* types (t208, t843, t1736, t1773, t2311, t3058, t3830, t4189, t9909, t15027) and six lineages (CC49, CC88, CC130, CC1956, sequence type (ST) 890, ST3033). The strains belonging to CC49 were PVL-positive whereas the CC130 isolates were PVL-negative [130]. Finally, a very recent study carried out in Austria analyzed the samples from 66 brown rats (*Rattus norvegicus*). Only one MRSA was isolated. It harbored the *mec*A gene along with the *bla*Z, *tet*(K), *tet*(M) and *erm*(A) genes, which confer resistance to tetracyline and erythromycin, and several virulence factors. This isolate was ascribed to *spa*-type t011 and CC398 [141].

Two studies have investigated the prevalence of *S. aureus* in wild hares. One of these studies was conducted in Portugal in 83 wild Iberian hares. Only three MRSA isolates were recovered all harboring the *mec*A and belonging to ST2855 and *spa*-type t1190. The isolates also carried the *bla*Z, *erm*C, *erm*B *erm*C, *mph*C *erm*C, *mph*C, *aac*(6′)-Ie-*aph*(2′′)-Ia genes conferring resistance to macrolides and aminoglycosides [142]. The second study was carried out in Germany and a total of 3 MRSA were isolated from 152 samples of European brown hares. All carried the *mec*C gene and were ascribed to CC130 and to the *spa*-types t10513 and t843 [51]. In both studies, the MRSA isolates were PVL-negative.

A case report described the isolation of one MRSA strain from a harbor seal admitted to a rehabilitation center in the Netherlands. This isolate belonged to CC9 and *spa*-type t1430, and showed resistance to penicillin, flucloxacillin, erythromycin, clindamycin and ciprofloxacin. Furthermore, it was PVL-negative [143].

Wild birds may also act as reservoirs of antibiotic-resistant bacteria, in particular, those living in close proximity to areas with high densities of livestock and inhabited by people. Furthermore, migratory birds can travel long distances in short periods of time carrying antibiotic-resistant bacteria; therefore, it is possible that wild birds can act as diffusers of antibiotic resistance. Rooks have been found to carry MRSA strains in Austria. Loncaric et al. (2014) studied 54 samples of a migratory population of rooks and 102 samples recovered from a non-migratory population. *mec*A-MRSA was found in five samples of migratory birds, also carrying resistance to ciprofloxacin, tetracycline, aminoglycosides and macrolides. All isolates were PVL-positive and were typed as CC1 and CC22 and spa-type t127 *and* t852 [144]. In another study conducted in birds from Austria, a large sample was screened for MRSA. From the 1,325 samples of wild birds analyzed, only three MRSA were detected [129]. In a study conducted with 16 birds of prey (*Buteo buteo*, *Strix aluco* and *Corvus corone*), one *S. aureus* was found in a common buzzard. The isolate presented phenotypic resistance to penicillin, tetracycline and chloramphenicol and belonged to CC30 *spa*-type t012 [146]. As described above, the *mec*C gene is widely distributed among wild mammals, and some studies have reported its presence among wild birds. Ninety-two white stork nestlings from Spain were sampled. *S. aureus* was found in 32 samples, of which three were methicillin resistant. One MRSA strain harbored the *mecC* gene and belonged to ST3061, which is a double-locus variant of ST130, and presented the *spa* type t843; while the other 2 MRSA harbored the *mec*A gene and belonged to the lineages CC398 *spa*-t011 and CC5-*spa*-t002. The remaining MSSA were ascribed to eight CCs (CC5, CC7, CC22, CC30, CC45, CC59, CC133 and CC398) and 18 spa-types (t1818, t1166, t6384, t6606, t571, t012, t688, t126, t209, t045, t015, t1945, t091, t3625, t774, t005, t216 and t14445) [145]. Ruiz-Ripa et al. (2019) carried out a study with 324 samples collected from healthy wild birds. *S. aureus* isolates were recovered from 15 wild birds, eight vultures and seven magpies, of which 13 were MRSA. Only one MRSA was *mec*A-positive (spa-type t011, CC398) and carried resistance to penicillin, cefoxitin, erythromycin, clindamycin, and tetracycline. Twelve MRSA isolates harbored the *mec*C gene and were typed as spa-type t843 and t1535 and ascribed to CC130. These *mec*C-positive isolates were susceptible for all non-β-lactams [53].

Finally, insects may also be colonized by staphylococci. *S. aureus* have also been found among flies in a study conducted by Schaumburg et al. (2016). In the same study, one MRSA was found among five MSSA isolated from flies. The MSSA belonged to spa-types t091, t362, t1535 and t2985 while the MRSA isolate harbored the *mec*A gene and showed characteristics of LA-MRSA (t011, CC398) [148].

As shown in the above studies, MRSA CC130 is the main lineage containing the *mec*C gene. Although the lineage CC130 was initially considered to be unique to animals, it displays a low host specificity since it has already been found among humans and in the natural environment. CC130 may have a zoonotic potential since it in was first discovered in cattle and is not very commonly associated with human infections [63]. Furthermore, transmission between different animal species belonging to CC130 has always been observed using complete genome sequencing [149]. Nevertheless, the *mec*C gene was also found among wild animals associated with other clonal lineages: CC1943 and CC2361. CC1943 have been also reported in bovine cattle in several countries and has been identified in human infections in Belgium also associated with *mec*C-MRSA strains [150]. *mec*A-MRSA among wild animals has been linked to several different clonal lineages: CC1, CC5, CC8, CC22, CC45, CC93 and CC398. Nevertheless, the most common clonal complexes found were CC1 and CC398. CC1 is common among humans and is considered a CA-MRSA. However, CC1 has been widely found among livestock, including pigs and mastitis in ruminants [151,152,153]. Furthermore, a similar strain of MRSA CC1 found in rooks has been found in humans in Romania [154]. MRSA CC398 was found among wild animals, mainly in wild boars, but also in rodents, storks and vultures. This clonal lineage is a livestock-associated lineage and has been detected in pigs and poultry [155,156]. The frequent detection of this clonal lineage among wild animals leads us to believe that CC398 may have originated from farm animals and has been spreading to the environment. Although being mainly associated with livestock species, CC398 has been isolated in humans in most European countries [157,158,159]. According to Price et al. (2012), MRSA CC398 might have evolved from the human strain MSSA CC398, and the jump to livestock might have caused it to acquire resistance to methicillin and tetracycline [160]. Therefore, neither CC1 and CC398 have pronounced host specificity for colonization and infection and have been detected colonizing multiple hosts, which may explain the frequent detection of these lineages among wild animals [132]. CC5 is a common and widespread lineage in humans but has been reported in companion animals and livestock [161,162]. The most frequently reported MRSA CCs collected from all continents are CC5, CC8, CC22, CC30 and CC45 [79]. Regarding the studies of European wildlife, CC5 MRSA and MSSA was found in animals mostly from Spain (storks, rodents and wild boars). Moreover, MSSA belonging to CC5 was the predominant *S. aureus* lineage found in surface waters which may indicate that this clonal lineage is widely disseminated in the natural environment of this country [103]. CC8 MRSA was found in a marten from Poland and in wild boars from Germany. CC8 has also been found to be particularly predominant in the hospital environment [163]. Although this was not a zoonotic lineage, CC8 was isolated from companion animals [164], horses [165], and livestock [152]. CC22 MRSA disseminated worldwide and are extremely common in Europe. Nevertheless, MRSA CC22 was only found in one wild animal species, namely in rooks from Austria. MSSA CC22 was also detected in storks and squirrels. CC45 and CC93 MRSA were found among wild boars in Germany. Both CC45 and CC93 are primarily associated with isolates from humans. CC93 was the predominant CC in humans in Australia and is usually associated with PVL positivity [166].

Twenty-one difference *spa*-types were found in *mec*C-MRSA strains isolated from European wildlife: t843, t3391, t978, t10751, t10893, t11015; t9111, t15312, t3256, t528, t1048, t3570, t6220, t17133, t2345, t8835, t16868, t5771, t10513, t4335 and t1535. The most predominant *spa*-type was t843 (fundamentally associated with CC130), which was found in eight strains within eleven studies reporting *mec*C-MRSA. This *spa* was found in a wide diversity of wild animals, namely, hedgehogs, hares, rodents, storks, rabbits, magpies and vultures, and also in livestock [50,167]. Although the detection of *mec*C-MRSA in humans is uncommon, *spa*-type t843 has been reported in humans and occasionally associated with zoonotic transmission [168,169]. *spa*-type t373 is one of the predominant types among *mec*C-MRSA strains isolated from humans, nevertheless, this *spa*-type has not been reported in *mec*C-MRSA from wildlife [168,170]. *mec*C-MRSA *spa*-types t978 and t3391 were reported in two different studies with hedgehogs, both studies reported a high frequency of t978, t3391 and t843 among these animals. Regarding the *mec*A-MRSA, 15 different *spa*-types were reported among wild animals: t386, t899, t011, t034, t1456, t1250, t015, t202, t008, t1190, t1635, t127, t852, t002 and t1535. *spa*-type t011 was the most frequently found among wildlife mostly associated with CC398. As mentioned previously, MRSA CC398 is known to be associated with livestock, particularly pigs. *spa*-type t127 was found in one *mec*A-MRSA and in three MSSA frequently associated with CC1. This *spa*-type has been found in humans and associated with livestock [171,172].

Some strains of *S. aureus* harbor genes encoding for PVL, which is one of the important cytotoxins produced by *S. aureus.* PVL is a marker of CA-MRSA since it is often present in these strains and rarely present in hospital isolates [173]. As expected, the genes encoding PVL were absent in all *mec*C-MRSA. On the contrary, several *mec*A-MRSA from three different animal species (martens, boars and rooks) were PVL-positive; yet, these MRSA strains belonged to clonal complexes linked with CA-MRSA.

Regarding antimicrobial resistance, *mec*C-MRSA were sensitive to non-β-lactam antimicrobials. These strains are usually associated with sensitivity phenotypes to the rest of non-β-lactam antimicrobials, although sporadic resistance to fluoroquinolones have been reported [174], the minimum inhibitory concentrations of oxacillin and cefoxitin are generally low compared to those reported in *mecA*-MRSA [175]. As for *mec*A-MRSA, the majority of the isolates presented resistance to other classes of antibiotics, including tetracyclines, fluoroquinolones, aminoglycosides and macrolides and lincosamides.

## 4. Conclusions

*S. aureus* is well distributed among European wildlife and presents a great diversity of genetic lineages, of which some have been previously associated with humans and livestock. Most MRSA strains isolated from wild animals are *mec*C-positive, corroborating the dissemination of this new mechanism of resistance in free-living animals. *mecC*-MRSA have been found in a wide range of other host species, from small mammals to wild birds from many European countries. Unlike most *mec*C-positive strains, the *mec*A-MRSA and MSSA isolates present resistance to other classes of antibiotics, rather than β-lactams, and several virulence factors. Considering that AMR are threatening our ability to treat common infectious diseases, wild animals may be overlooked as a transmission vector of antibiotic resistant bacteria. These studies show the importance of wildlife as reservoirs of *S. aureus* and MRSA with a possible role in transmission and propagation of these strains.

## Figures and Tables

**Table 1:** Molecular typing methods for *S. aureus.*

Typing Methods	Principle	Reference
***agr* typing**	Amplification of the hypervariable segment	[86]
***spa* typing**	Amplification and sequencing of the 24 bp polymorphic zone X of the *spa* gene	[87]
**Mutilocus sequence typing (MLST)**	sequence analysis of the allelic variants of the seven housekeeping genes	[88]
**SCC*mec* typing**	Analysis of the structure of SCC*mec* region	[89]
**Pulsed-field gel electrophoresis (PFGE)**	Macro-restriction profiling based on the digestion of DNA with restriction enzymes	[90]
**Random Amplification of Polymorphic DNA (RAPD)**	Unspecific binding with polymorphism of the whole chromosome	[91]
**Multilocus variable-number tandem repeat (VNTR) analysis (MLVA)**	Polymorphism of tandemly repeated DNA sequences	[92]
**Multiple locus VNTR fingerprinting (MLVF)**	polymorphism of tandemly repeated DNA sequences	[93]
**Genome-scale DNA microarrays**	Hybridization with genes on the chromossome	[94]
**Whole genome sequencing (WGS)**	Genome-wide variations	[95]
**Matrix-Assisted Laser Desorption Ionization - Time of Flight (MALDI-TOF) Mass Spectrometry (MS)**	Generation of mass-spectral fingerprints	[96]

**Table 2:** Animal species, location of isolation and genetic lineages of S. aureus and MRSA isolated from European wild animals.

Animal	Location	MRSA/MSSA (Number of Isolates)	Clonal Lineages		Reference
			*spa*-Type	ST/CC	
**Hedgehog**	**Sweden**	***mec*C-MRSA** **(35)**	**t843, t3391, t978, t10751 t10893, t11015; t9111, t15312**	**CC130, CC2361**	[121]
	Poland	**MSSA** (1)	n.d.	n.d.	[126]
	Austria	***mec*C-MRSA** (1)	**t3256**	**CC130**	[51]
	Denmark	***mec*****C-MRSA** (114)	**t528, t843, t1048, t3256, t3570, t6220, t17133, t978, t2345, t3391, t8835, t16868**	**CC130** **CC1943**	[134]
	Spain	***mec*A-MRSA** (1)	**t386**	**CC1**	[54]
	Sweeden	***mec*C-MRSA** (2)	**t843** **, t5771**	**CC130**	[135]
Wild Boar	Spain	MSSA (11)	t1535, t7174, t1534, t6386, t3750, t11230	CC130, CC5, CC522, CC425, ST2328	[54]
	Portugal	***mec*A-MRSA** (1) MSSA (29)	**t899**, t3750, t1533, t286, t14312, t14311, t10668, t3583, t3750, t11230, t10712	**CC398**, ST3220, ST1, ST3224, ST3223, ST3222, ST133, ST2328, ST1643	[122]
	Germany	***mec*A-MRSA** (28)	**t011, t034, t1456, t1250, t015, t202, t008**	**CC398, CC45, CC93, CC8**	[136]
	Spain	***mec*A-MRSA (1)** MSSA (50)	**t011**, t3750, t16741, t3583, t742, t11232, t6292, t11212, t002, t1094, t127, t843, t12923, t208, t1951, t1200, t073, t12827, t16740, t548, t3293	**CC398**, CC133, CC425, CC5, CC1, CC130, CC49, CC88, CC97	[137]
	Germany	MSSA (41)	t127, t091, t14149, t021, t1773, t11226, t1181, t7674, t12042, t10856, t3369, t15002, t6902, t15001, t15000, t3583, t742, t14999, t571	ST1, ST7, ST30, ST890, ST3237, ST3238, ST3369, ST425, ST3255, ST133, ST804	[138]
	Spain	***mec*A-MRSA** (7)	**t011, t127**	**CC398**	[132]
	Spain	MSSA (126)	t098, t127, t607, t1407, t2601, t11223, t548, t2516, t7174, t11210, t11214, t11219, t084, t11218, t6220, t3583, t10476, t11220, t189, t034,t742, t6909, t11222, t11225, t11232, t10712, t3750, t11227, t11230, t11229, t359, t11209, t11502, t015, t6384, t011	ST1, ST5, ST15, ST96, ST130, ST133, ST188, ST398, ST425, ST1643, ST2328, ST2641, ST2672, ST2675, ST2678, ST2681, ST2682, ST2729,	[139]
Rodent	Spain	***mec*C-MRSA** (2) MSSA (11)	**t1535**, t120, t12365, t12752, t9303, t3750, t12363, t12364	**CC130**, CC5, CC1956, ST2328 ST2766, ST2767	[140]
	Slovakia	***mec*A-MRSA** (3) MSSA (4)	n.d.	n.d.	[125]
	Germany	***mec*C-MRSA** (1) 39 MSSA	**t843**, t208, t4189, t1773, t2311, t15027, t3058	**CC130**, CC49, ST890, CC88, CC1956	[130]
	Austria	***mec*A-MRSA** (1)	**t011**	**CC398**	[141]
Deer	Spain	MSSA (4)	t1535	CC130	[54]
	Germany	MSSA (1)	t15473	CC425	[133]
	Spain	MSSA (54)	t098, t127, t11223, t548, t11210, t342, t2678, t11215, t571, t1077, t6386, t6909, t11208, t11212, t11228, t11231, t528, t1534, t3576, t742, t11211, t11226, t11233, t015, t11217	ST1, ST5, ST30, ST133, ST350, ST398, ST425, ST522, ST2640, ST2671, ST2681	[139]
Hare	Portugal	***mec*A-MRSA** (3)	**t1190**	**ST2855**	[142]
	Germany	***mec*C-MRSA** (3)	**t10513, t843**	**CC130**	[51]
Marten	Poland	***mec*A-MRSA** (1) MSSA (2)	**t1635**	**CC8**	[126]
Red Foxe	Poland	MSSA (2)	n.d.	n.d.	[126]
Otter	Austria	***mec*C-MRSA** (1)	**t4335**	**CC130**	[51]
Shrew	Germany	MSSA (5)	t9909, t1125, t11225	ST3033 CC5, CC425	[130]
Rabbit	Spain	***mec*C-MRSA** (3)	**t843**	**CC130**	[54]
Mouflon	Spain	MSSA (4)	t6056, t11233	CC133, ST3237	[54]
Ibex	Spain	MSSA (36)	t002, t1736, t3369, t528, t843, t1535, t3750, t11501, t11221, t7229, t11216, t528	ST5, ST130, ST425, ST581, ST2328, ST2637, ST2639, ST2673	[139]
	Spain	MRSA (2)	t011, t1451	CC1, CC398	[132]
Squirrel	Germany	MSSA (4)	t208, t307, t528	CC49, CC22, ST4310	[133]
Beaver	Germany	MSSA (1)	t3058	CC1956	[133]
Seal	The Neatherlands	MRSA (1)	t1430	CC9	[143]
Bat	Germany	MSSA (1)	t164	CC20	[133]
Rook	Austria	***mec*A-MRSA** (5)	**t127, t852**	**CC1, CC22**	[144]
Stork	Spain	***mec*C-MRSA** (1) ***mec*A-MRSA** (2) MSSA (35)	**t843, t002, t011**, t1818, t1166, t6384, t6606, t571, t012, t688, t126, t209, t045, t015, t1945, t091, t3625, t774, t005, t216, t14445	**CC130** **CC5** **CC398** CC7, CC22, CC30, CC45, CC59, CC133	[145]
Eagle	Germany	MSSA (1)	t1422	CC692	[133]
Kite	Germany	MSSA (1)	t14745	CC692	[133]
Magpie	Spain	***mec*C-MRSA** (7)	**t843**	**CC130**	[53]
Vulture	Spain	***mec*C-MRSA** (5) ***mec*A-MRSA** (1) MSSA (2)	**t843, t011, t1535**, t267	**CC130, CC398**, C97	[53]
	Spain	MSSA (2)	t7304	ST133	[139]
Buzzard	Portugal	MSSA (1)	t012	CC30	[146]

Abbreviations: ST: sequence type; CC: clonal complex. Note: *spa*-types and CC/ST in bold correspond to MRSA isolates.
